# Are households with under-five children in Nigeria socioeconomically disadvantaged?

**DOI:** 10.1371/journal.pgph.0002616

**Published:** 2024-01-30

**Authors:** Ashwini Sunil Deshpande, Osondu Ogbuoji

**Affiliations:** Duke Center for Policy Impact in Global Health, Duke Global Health Institute, Durham, North Carolina, United States of America; Pennsylvania State University, UNITED STATES

## Abstract

Although the sociodemographic and economic contributors to under-five mortality are well established, very little research has been done to assess the levels of disadvantage under-five children in Nigeria face along these dimensions. Nigeria has the second-highest under-five mortality rate (U5MR) in the world (111 deaths per 1000 live births) and contributed to the highest number of annual under-five deaths globally in 2020 (844,321 deaths). The country has also implemented several decades of policy interventions to reduce under-five mortality by improving sociodemographic and economic conditions at the household level. In this paper, we assess the sociodemographic and economic disadvantages that households with children under-five face compared to other households and discuss the implications for health policy. Using the Nigeria Living Standard Survey 2018–19, we conducted a bivariate analysis to compare the sociodemographic and economic characteristics of households with and without under-five children. We performed independent samples t-test and proportions test to assess whether these sociodemographic and economic factors were significantly different for both groups. We found that households with under-five children typically had larger sizes (6.6 vs. 3.6), lower mean adult age (36.5 vs. 45.3), and male household heads (91.3% vs. 71.5%) than households without under-five children. Furthermore, households with under-five children were less likely to have access to improved drinking water (77.2% vs. 86.0%) and sanitation sources (54.0% vs. 61.9%) than those without under-five children. Despite having more adult working members, 71.2% of households with under-five children lived below the poverty line compared to 37.7% of other households. Although their total consumption expenditure was lower than households without under-five children, they spent a higher proportion of their expenditure on health care and were at a higher risk of experiencing catastrophic health expenditure. Our study has shown that households with children under five are disproportionately disadvantaged than other households in Nigeria. The households with under-five children are larger, younger, and poorer than those without children. We also show a wide variation in the proportion of households with children under five by state. Any efforts to reduce under-five mortality and morbidity in Nigeria should recognize these sociodemographic and economic differences.

## Introduction

Under-five mortality remains a major concern in Nigeria. In 2020, Nigeria recorded the second-highest under-five mortality rate (U5MR) in the world (111 deaths per 1000 live births) and contributed to the highest number of annual under-five deaths globally (844,321 deaths) [1]. under-five mortality (U5M) worsened during the COVID-19 pandemic, with increased newborn deaths from interrupted health services, almost reversing a decade of progress in reducing preventable under-five mortality [2]. The factors contributing to high U5M in Nigeria have been established. These include access to essential health services, access to clean water and sanitation, household wealth (and poverty), residence in urban vs. rural areas, and other household socio-demographic characteristics like age and education of the household head.

Over the past decades, based on this body of evidence, policy solutions to address U5M have targeted these sociodemographic and economic determinants. It is, therefore, important to assess households with children under five currently along these dimensions and to see if they fare better or worse than the average Nigerian household. Characterizing important aspects of the lived experiences of households with children under five is the first step toward understanding their unique challenges and improving their access to life-saving healthcare. Previous studies indicate that children in Nigeria are disproportionately affected by poverty [3, 4]. For example, Ogwumike and Ozughalu found that in Nigeria, 70% of children were living in overall poverty, defined as living below two-thirds of the national average total household expenditure per capita [4].

In this paper, we use the Nigeria Living Standard Survey (NLSS) 2018–19 to assess how much of a disadvantage under-five children face by comparing their household socioeconomic factors with other households that do not have under-five children. In addition to characterizing the peculiar nature of households with under-five children, the findings highlight some challenges these households face in ensuring the health and well-being of their children.

## Methods

### Data

We analyzed data from the Living Standard Survey (NLSS) implemented in Nigeria from September 2018 to September 2019 [5]. It is the first large-scale cross-sectional household survey in Nigeria since 2011 that focuses on measuring the population’s living standard. The data is collected at the individual, household, and community levels and provides representative estimates for the 36 states and the Federal Capital Territory (FCT), Abuja. The 2018/19 NLSS sample covered 22,110 households surveyed in 60 enumeration areas (EA) per state. However, the sample from Borno State was non-representative due to a higher level of nonresponse, possibly due to prolonged terror conflicts. Therefore, we excluded Borno State from our analysis.

NLSS has information on various socioeconomic and demographic factors, including all household members’ education and health status, household consumption expenditure, assets, housing conditions, and access to community infrastructure. In addition, the survey is specifically designed to measure welfare and poverty. These features of NLSS enable us to identify sociodemographic differences and similarities in households with and without under-five children. We used deidentified data and did not have access to any personally identifying information of people included in the datasets. Data were accessed between August 26, 2022, and Dec 23, 2022, for research purposes.

### Study variables

Households with at least one child below five years of age were categorized as households with under-five children. These households were compared with two other groups of households: (i) households without under-five children and (ii) all households in the country. We described the identifying social, demographic, and economic features of these groups of households (Table 1).

**Table 1:** Variable definitions.

Variables	Definitions
Sociodemographic characteristics	
Household size	Total number of household members
Average age of adult household members	Average age of household members above 18 years
Average percentage of working adults to total adult members in the household	Ratio of working adults to total adult members in the household Working adult member is defined as any adult (>18 years) household member who has worked for someone who is not a member of this household (for example, an enterprise, company, the government or any other individual), worked on a farm owned or rented by a household member, worked on his/her own account or in family business enterprise or worked as a trainee or apprentice in any career-oriented skills in 7 days before the survey.
Households with female heads	Households with females as the household head
Households with household head with schooling	Households with household head who did not attend school
Households in rural areas	Households located in rural Nigeria
Households with an improved source of water during the rainy season	The water sources are likely to be of suitable quality or "improved", are: a piped water supply into the dwelling; piped water to a yard/plot; a public tap/standpipe; a tube well/borehole; a protected dug well; a protected spring; and rainwater. Water sources that are "unimproved" are: an unprotected dug well; an unprotected spring; a cart with a small tank/drum; a water tanker truck; and surface water [6]
Households with an improved source of sanitation	A sanitation facility is considered improved if it hygienically separates human excreta from human contact. The types of technology that are likely to meet this criterion are: flush to the piped sewer system; flush to septic tank; flush/pour flush to the pit; composting toilet; VIP latrine; pit latrine with a slab. Types of sanitation facilities that are not likely to meet the criterion are: flush/pour flush elsewhere; pit latrine without a slab/open pit; bucket; and a hanging toilet [6]
Variables	Definitions
Average annual household consumption expenditure per capita	Average annual household consumption expenditure per capita spatially and temoraly adjusted by the World Bank using the survey information
Average annual household healthcare expenditure per capita	Average annual household healthcare expenditure per capita computed by the World Bank using the survey information
Proportion of healthcare expenditure to consumption expenditure	Average annual household healthcare expenditure per capita as a percentage of average annual household consumption expenditure per capita
**Poverty measures (at the World Bank International Poverty Line) [7]**	
Gross poverty headcount	Households whose average annual consumption expenditure per capita gross of average annual healthcare expenditure per capita is below the poverty threshold are considered to be living in gross poverty
Net poverty headcount	Households whose average annual consumption expenditure per capita net of average annual healthcare expenditure per capita is below the poverty line are considered to be living in net poverty
Medical impoverishment	The difference between the net poverty headcount and the gross poverty headcount gives the medical impovershing effect (headcount).
Gross poverty gap	The gross poverty gap is estimated as the amount by which the gross poor households fall short of reaching the poverty threshold
Net poverty gap	The net poverty gap is estimated as the amount by which the net poor households fall short of reaching the poverty threshold
**Financial risk protection measures [7]**	
Catastrophic health expenditure (CHE) at 5%, 10% and 25% threshold	A household is considered to experience CHE when its average annual healthcare expenditure per capita exceed the appropriate threshold percent of the household’s average annual consumption expenditure per capita. In accordance with the sustainable development goals (SDG) we use three thresholds– 5%, 10%, and 25%.
Overshoot at 5%, 10% and 25% threshold	Overshoot captures the average degree by household’s average annual healthcare expenditure per capita, as a proportion of its average annual per capita consumption expenditure, exceeds the threshold
Mean positive overshoot (MPO) at 5%, 10% and 25% threshold	MPO is estimated as the average overshoot only for only those households that experience CHE.

Sociodemographic factors assessed include household location (rural/urban), gender of household head (male/female), schooling status of household head (attended school/not attended school), household size, number of adult working members, and availability of improved water and sanitation sources in the household. We defined improved sources of water and sanitation in accordance with the UNICEF guidelines [6].

Economic factors assessed include poverty and catastrophic health expenditure measures. Poverty measures included gross poverty headcount (i.e., the total number of people living below the poverty threshold), the net poverty headcount (the estimated total number of people living below the poverty threshold after excluding health expenses), and the poverty gap (i.e., a measure of severity that indicates how far below the poverty threshold a household is) [7]. To estimate the poverty measures, we compared the household’s annual consumption expenditure per capita with the World Bank’s extreme poverty threshold of US$ 1.9 per person per day (US$ 694 per person per year, equivalent to NGN 212,765 per person per year in 2019). Households with annual consumption expenditure per capita below the poverty threshold were included in the gross poverty headcount. A household was defined as net poor when the household’s total annual consumption expenditure per capita net of annual health expenditure per capita fell below the poverty threshold. The difference between the net poor and the gross poor headcount was defined as medical impoverishment (i.e., a new case of poverty attributed to health expenditure). The poverty gap was then estimated as the amount by which the gross and net poor households fell short of reaching the poverty line. In S2 Appendix, we estimated these poverty measures based on Nigeria’s national poverty threshold (NGN 137,430 per person per year in 2019, equivalent to 2019 US$ 448).

Next, we compared households with and without under-five children on catastrophic health expenditure (CHE) measures [7]. A household experiences CHE when the cost of using health care exceeds the appropriate threshold percent of the household’s income or consumption expenditure. We measured four CHE thresholds– 5%, 10%, and 25%. CHE intensity was estimated using overshoot and mean positive overshoot (MPO) measures. Overshoot was defined as the average degree by which a household’s annual health expenditure per capita, as a proportion of a household’s annual consumption expenditure per capita, exceeds the CHE threshold. While MPO was estimated as the average overshoot only for households that experience CHE. We used the spatially and temporally adjusted annual household consumption expenditure per capita and household health expenditure per capita variables computed by the World Bank from the survey information for this analysis.

### Statistical analysis

We used independent samples t-test to assess group differences in means of continuous variables and a proportions test to determine group differences for binary variables. We accounted for stratification at the state level and clustering at the enumeration area to estimate standard errors correctly. We also applied analytical weights to obtain nationally representative point estimates.

### Ethics statement

The study was approved by the Duke Institutional Review Board #2022–0521.

## Results

Our analytical sample consisted of 21,580 households, excluding those surveyed in Borno state, because of the chronic conflict experienced in the state over the decade and its impact on data quality. Half of the households (n = 10,918) had at least one child below five years of age, and the remaining (n = 10,662) had no children below five years (Table 2).

**Table 2:** Sociodemographic characteristics of households with and without children below five years of age.

	Households with children below 5 years	Households without children below 5 years	All households	difference (Column [1]- Column [2]) = 0 P>|t|^1^
	[1]	[2]	[3]	[4]
**Sociodemographic characteristics**				
Number of households in the sample (unweighted)	10,918	10,662	21,580^2^	
1. Household size	6.6	3.6	5.1	<0.001
2. Average age of adult household members	36.5	45.3	41.0	<0.001
3. Average percentage of working adults to total adult members in the household	77.7	74.0	75.8	<0.001
4. Percentage of households with female heads	8.7	28.5	18.8	<0.001
5. Percentage of households with household head with schooling	86.8	80.6	83.7	<0.001
6. Percentage of households in rural areas^3^	65.9	55.2	60.4	<0.001
7. Percentage of households with an improved source of water during the rainy season	77.2	86.0	81.7	<0.001
8. Percentage of households with an improved source of water during the dry season	72.8	80.2	76.6	<0.001
9. Percentage of households with an improved source of sanitation	54.0	61.9	58.0	<0.001

Notes

1. We used independent samples t-test to assess group differences in means of continuous variables [1–3] and a proportions test to determine group differences for binary variables [4–9]. Both, the independent samples test and proportions test indicate differences between columns 1 and 2.

2. Due to a higher level of nonresponse, the sample from Borno State is non-representative, hence excluded from the analysis.

3. The estimates indicate the percentage of households with and without under-five children in rural areas

Households with under-five children had, on average, 6.6 household members, almost double the size of households without children under-five (3.6 household members) and higher than the national average (5.1 household members) (Table 2). Adults in under-five households were younger, with an average age of 36.5 years, compared to 45.3 years in households without under-five children. The proportion of adult working members to total household number was slightly higher in under-five households (77.7% vis-a-vis 74.0%), and they were more likely to have a male household head (8.7% female-headed households compared to 28.5% among households without under-five children). A higher proportion of households with under-five children had a household head with some schooling (86.8% vis-à-vis 80.6%). Furthermore, households with children under five were more likely to be located in rural areas than households without (65.9% vs. 55.2%).

Regarding household living conditions, including drinking water and sanitation sources, households with under-five children fare worse than those without under-five children (Table 2). Improved sources of drinking water are those that are likely to be protected from outside contamination. A significantly lower proportion of households with under-five children (77.2%) were likely to access improved drinking water sources than those without under-five children (86.0%). Similarly, improved sanitation sources hygienically separate human waste from human contact. However, households with under-five children were significantly less likely to have improved sanitation sources than those without under-five children (54.0% vis-à-vis 61.9%).

Some states had a higher proportion of households with under-five children than others. In states like Bauchi, Katsina, Kebbi, Niger, Yobe, and Zamfara, more than 70% of households had children under five years of age. Whereas less than 35% of households in Bayelsa, Cross River, and Lagos had under-five children (S1 Appendix).

Households with children under five earn less yet pay more for health. The average annual consumption and healthcare expenditure per capita of households with under-five children were significantly lower than those without children under five (Table 3). On average, households with under-five children spent half of the amount spent by those without under-five children (NGN 181,783/US$ 593 compared to NGN 325,400/US$ 1,061 per year). Similarly, households with under-five children spent only NGN 10,733 (US$ 35) on health care per year, about three-fifth of the amount spent by those without under-five children (NGN 17,518/US$57). However, the ratio of healthcare expenditure to total expenditure was higher for households with under-five children (6.3% vis-à-vis 5.8%).

**Table 3:** Financial risk measures of households with and without children below five years of age.

	Households with children below 5 years	Households without children below 5 years	All households	difference (Column [1]- Column [2]) = 0 P>|t|^1^
	[1]	[2]	[3]	[4]
1. Average annual consumption expenditure per capita, NGN (USD^2^)	181,783.1 (592.5)	325,400.0 (1060.6)	255,434.1 (832.6)	<0.001
2. Average annual health expenditure per capita, NGN (USD^2^)	10,732.9 (35.0)	17,518.1 (57.1)	14,212.6 (46.3)	<0.001
3. Annual health expenditure per capita (as a percentage of total annual consumption expenditure per capita)	6.3	5.8	6.0	<0.001
**Poverty measures (at the World Bank International Poverty Line)** ^**3**^				
4. Gross poverty headcount percent	71.2	37.7	54.0	<0.001
5. Net poverty headcount percent	74.4	42.1	57.8	<0.001
6. Medical impoverishment percent	3.1	4.4	3.8	<0.001
7. Gross poverty gap in NGN (USD^2^)	62,196.1 (202.7)	25,809.1 (84.1)	43,535.8 (141.9)	<0.001
8. Net poverty gap in NGN (USD^2^)	68,644.5 (223.7)	30,395.9 (99.1)	49,029.5 (159.8)	<0.001
**Financial risk protection measures**				
9. Percentage of households with catastrophic health expenditure at 5% threshold^4^	42.5	34.4	38.4^	<0.001
10. Percentage of households with catastrophic health expenditure at 10% threshold	21.1	18.9	20.0	0.001
11. Percentage of households with catastrophic health expenditure at 25% threshold	3.1	4.4	3.8	<0.001
12. Overshoot at 5% threshold^5^	3.2	3.3	3.2^^	0.462
13. Overshoot at 10% threshold	1.7	2.0	1.8	<0.001
14. Overshoot at 25% threshold	0.3	0.5	0.4	<0.001
15. Mean positive overshoot at 5% threshold^5^	7.5	9.5	8.5^^^	<0.001
16. Mean positive overshoot at 10% threshold	7.9	10.5	9.2	<0.001
17. Mean positive overshoot at 25% threshold	9.1	12.0	10.8	0.002

Notes

1. We used independent samples t-test to assess group differences in means of continuous variables [3,4,5,6,7,11,12,13,14,15,16] and a proportions test to determine group differences for binary variables [1,2,8,9,10]. Both, the independent samples test and proportions test indicate differences between columns 1 and 2.

2. 1 NGN = 306.8 USD

3. The World Bank Internal Poverty Line is defined at 1.9 a day per person. It translates to NGN 212,765 per person annually at 1 NGN = 306.8 USD.

4. ^ indicates that 38.4% of households in Nigeria spent more than 5% of annual household consumption expenditure per capita on health care.

5. ^^ indicates that on average health care payments were 3.2 percentage points higher than the 5% CHE threshold. However, for those households experiencing CHE, health care payments were 7.5 percentage points higher than the 5% threshold.

Poverty was a significant identifying factor in households with under-five children (Table 3). Around 71.2% of households with under-five children were identified as gross poor, where poverty was defined at the World Bank’s international poverty line. In contrast, only 37.7% of households without under-five children were gross poor. The poverty gap was significantly higher for households with under-five children than those without children. On average, gross poor households with under-five children spent NGN 62,196 (US$ 203) per person less than the poverty threshold. However, the poverty gap experienced by gross poor households without under-five children was NGN 25,809 (US$ 84) per person less than the poverty threshold. The net poverty count was also higher among households with under-five children. Around 74.4% of households with under-five children were net poor, whereas that percentage was only 42.1% among households without under-five children. As a result, the net poor households with under-five children fell short of reaching the poverty threshold by NGN 68,645 (US$ 224) per person. On the other hand, the net poor households without under-five children fell short by only NGN 30,396 (US$ 99) per person.

We also estimated poverty measures using Nigeria’s national poverty line of NGN 137,430 per person per year in 2019 (equivalent to 2019 US$ 448) (S2 Appendix). Around 42.1% of households with under-five children were below Nigeria’s national poverty line, about three times more than in households without under-five children (16.1%). On average, poor households with under-five children spent NGN 18,475 (US$ 60) per person less than the national poverty line. In contrast, the poverty gap for poor households without under-five children was NGN 5,695 (US$ 19) per person, a third of the poverty gap experienced by households with under-five children.

Households with under-five children were also more likely to experience catastrophic health expenditure at 5% and 10% thresholds, but not at 25% (Table 3). Around 42.5% of households with under-five children spent more than 5% of total household expenditure on health care. By contrast, only 34.4% of households without under-five children spent more than 5% on health care. Similarly, a slightly higher proportion of households with under-five children (21.1%) spent more than 10% of their expenditure on health than those without under-five children (18.9%). However, at a higher CHE threshold of 25%, households with under-five children were less likely to experience catastrophic health expenditure (3.1% vs. 4.4%).

The overshoot and MPO estimates indicate the intensity of CHE (Table 3). On average, for households with under-five children, healthcare payments were 3.2 percentage points higher than the 5% CHE threshold. However, for those households with under-five children experiencing CHE, healthcare payments were 7.5 percentage points higher than the 5% threshold. Taken together, the overshoot and MPO estimates imply that, on average, households with children under five spent about 8.2% of total expenditure on healthcare, while in the under-five households that experience CHE, the average spending on healthcare was 12.5%.

## Discussion

After decades of public health interventions to improve children’s health in Nigeria, under-five children continue to face significant disadvantages along important sociodemographic and economic dimensions. Our results show that compared to other households, the households with under-five children in Nigeria are larger, poorer, more likely to be in a rural region, more exposed to the financial risk of seeking care, and have less access to improved drinking water and sanitation. Despite having more adult working members in the household, most households with under-five children live below the poverty line. Although their total consumption expenditure is lower than households without under-five children, they spend a higher proportion of their expenditure on health care. As a result, households with under-five children are at a higher risk of experiencing catastrophic health expenditures. Moreover, households with under-five children are less likely to have improved sanitation and access to safe drinking water than households without under-five children.

These findings raise important policy questions and have important policy implications. For example, improved access to water and sanitation are essential for good health. Why, then, do households with under-five children have reduced access to these services in a country where diarrheal diseases are a major cause of under-five mortality? The Nigerian Health Insurance Basic Minimum Package of Health Services includes treatment for diarrhea diseases, but there is no provision for preventative measures such as providing safe drinking water and sanitation for all, particularly for households with under-five children. Furthermore, previous research indicates that financial risk prevents households from seeking appropriate health care, and developing poor health-seeking behaviors that significantly contribute to child morbidity and mortality [8]. By having higher financial risk exposure, households with children under five are more likely to forgo care or adopt poor health seeking behaviours, making it difficult to make significant progress on child mortality prevention.

Our finding that households with children are predominantly male-headed is important because previous studies show a difference in the health-seeking behavior for childhood illnesses between households with female and male heads. Female-headed households are more likely to seek health care for childhood illnesses than male-headed households [9]. Therefore, interventions promoting children’s health in Nigeria should also target fathers or male members of the households to ensure the child’s well-being. Finally, our results highlighting the geographical location of households with under-five children in Nigeria (rural/urban, regions, and states) can help with program planning and implementation. For example, states like Yobe, Zamfara, and Kebbi, with over 70% of households having at least one under-five child, will require a different health benefits package compared to states like Bayelsa, Cross River, and Lagos, with less than 35% of households having children under five.

The strength of this study is that we use NLSS 2018–19, which was explicitly designed to capture welfare and poverty in Nigeria. However, a limitation of our study is that our analysis is limited to a set of sociodemographic and economic factors available in the NLSS 2018–19 dataset. Moreover, the information on sociodemographic and economic factors comes from the pre-COVID period, and evidence suggests that the COVID-19 pandemic has impacted the health and economics of Nigerian households. For example, a recent study analyzing data from 35 lower and middle-income countries, including Nigeria, found that two-thirds of households with under-five children lost income during the pandemic [10]. So, the current economic situation of the under-five households might be worse than that found in this analysis. Furthermore, our study did not control for the age of the child and disease severity within the groups; rather, we grouped households as having under-five children or not. While our approach addresses the main question of this paper, our results should be interpreted with caution as we do not highlight the differential onset and subsequent effect of childhood illness on the family at different ages. For example, within the under-five group, a family with a newborn suffering from neonatal sepsis will have a different experience from a family with a four-year-old suffering from non-complicated malaria.

In summary, this study highlights the peculiar nature of households with children under five and the unique challenges they face. Poverty and high catastrophic health spending are identifying characteristics of households with children under five.

## Conclusion

Our study has shown that households with children under five are disproportionately disadvantaged than other households in Nigeria; they are larger, younger, and poorer than those without children under five. We also show that poverty and high catastrophic health spending are identifying characteristics of households with children under five and that there exists a wide variation in the proportion of households with children under five by state. Any efforts to reduce under-five mortality and morbidity in Nigeria should recognize these sociodemographic and economic differences. Future research must also investigate how policies can be adapted to maximize child health outcomes and financial risk protection for the most vulnerable households.

## Supporting information

S1 Appendix Percentage of households with children under five years of age in each state. (DOCX)

S2 Appendix Financial risk measures of households with and without children below five years of age. (DOCX)
