# Supplementary material for: Are households with under-five children in Nigeria socioeconomically disadvantaged?
Source: PLOS Glob Public Health. 2024 Jan 30;4(1):e0002616. doi: 10.1371/journal.pgph.0002616 (PMC10826941; doi:10.1371/journal.pgph.0002616)
Supplement: S1 Appendix — (DOCX) [file pgph.0002616.s001.docx]

S1 Appendix: Percentage of households with children under five years of age in each state

| State | Percentage of under-five households to all households | | |
| --- | --- | --- | --- |
|  | Total | Rural | Urban |
| Abia | 37.0 | 34.5 | 42.7 |
| Adamawa | 57.9 | 57.9 | 57.6 |
| Akwa Ibom | 41.8 | 41.4 | 43.9 |
| Anambra | 46.2 | 43.5 | 48.1 |
| Bauchi | 72.7 | 72.3 | 75.4 |
| Bayelsa | 33.1 | 31.0 | 39.9 |
| Benue | 45.1 | 46.2 | 41.3 |
| Cross River | 34.5 | 36.9 | 23.4 |
| Delta | 43.2 | 48.2 | 34.0 |
| Ebonyi | 48.8 | 49.2 | 42.7 |
| Edo | 41.2 | 43.8 | 38.4 |
| Ekiti | 34.8 | 37.7 | 33.9 |
| Enugu | 36.3 | 35.2 | 41.3 |
| Gombe | 67.1 | 68.0 | 62.8 |
| Imo | 36.0 | 38.1 | 18.0 |
| Jigawa | 75.8 | 76.3 | 73.2 |
| Kaduna | 63.0 | 63.5 | 62.2 |
| Kano | 68.8 | 71.1 | 63.3 |
| Katsina | 72.5 | 72.4 | 73.1 |
| Kebbi | 70.6 | 69.8 | 78.2 |
| Kogi | 48.4 | 50.0 | 45.8 |
| Kwara | 45.0 | 45.7 | 44.1 |
| Lagos | 31.6 | 48.1 | 30.3 |
| Nasarawa | 66.3 | 67.8 | 61.2 |
| Niger | 73.3 | 73.6 | 72.3 |
| Ogun | 35.7 | 35.6 | 35.8 |
| Ondo | 38.0 | 36.6 | 38.9 |
| Osun | 37.9 | 30.3 | 40.6 |
| Oyo | 39.0 | 36.0 | 40.1 |
| Plateau | 56.7 | 58.6 | 52.2 |
| Rivers | 41.1 | 43.5 | 35.1 |
| Sokoto | 67.1 | 66.3 | 70.7 |
| Taraba | 58.0 | 58.7 | 50.0 |
| Yobe | 75.8 | 76.4 | 73.6 |
| Zamfara | 70.7 | 72.7 | 61.5 |
| FCT | 58.5 | 64.5 | 51.4 |
