# Supplementary material for: Are households with under-five children in Nigeria socioeconomically disadvantaged?
Source: PLOS Glob Public Health. 2024 Jan 30;4(1):e0002616. doi: 10.1371/journal.pgph.0002616 (PMC10826941; doi:10.1371/journal.pgph.0002616)
Supplement: S2 Appendix — (DOCX) [file pgph.0002616.s002.docx]

S2 Appendix: Financial risk measures of households with and without children below five years of age

|  | **Households with children below 5 years** | **Households without children below 5 years** | **All households** | **difference (Column [1]- Column [2])=0 P>\|t\|^1^** |
| --- | --- | --- | --- | --- |
|  | [1] | [2] | [3] | [4] |
| **Poverty measures (at the National Poverty Line)^2^** | | | | |
| 1. Gross poverty headcount percent | 42.1 | 16.1 | 28.8 | <0.001 |
| 2. Net poverty headcount percent | 47.2 | 19.0 | 32.8 | <0.001 |
| 3. Medical impoverishment | 5.1 | 3.0 | 4.0 | <0.001 |
| 4. Gross poverty gap in NGN (USD^3^) | 18,475.4 (60.2) | 5,695.2 (18.6) | 11,921.3 (38.9) | <0.001 |
| 5. Net poverty gap in NGN (USD^3^) | 21,742.7 (70.9) | 7,206.7 (23.5) | 14,288.2 (46.6) | <0.001 |

**Notes:**

1. We used independent samples t-test to assess group differences in means of continuous variables [4,5] and a proportions test to determine group differences for binary variables [1,2,3]. Both, the independent samples test and proportions test indicate differences between columns 1 and 2.
2. The National Poverty Line is defined at NGN 137,430 per person per year.
3. 1 NGN= 306.8 USD
